# The burden of depressive disorders in South Asia, 1990–2016: findings from the global burden of disease study

**DOI:** 10.1186/s12888-018-1918-1

**Published:** 2018-10-16

**Authors:** Felix Akpojene Ogbo, Sruthi Mathsyaraja, Rajeendra Kashyap Koti, Janette Perz, Andrew Page

**Affiliations:** Translational Health Research Institute (THRI), School of Medicine, Western Sydney University, Locked Bag 1797, Penrith, NSW 2571 Australia

**Keywords:** Depressive disorders, Depression, Global Burden of Disease, South Asia

## Abstract

**Background:**

Globally, depressive disorders are one of the most common forms of mental illness. Using data from the most recent Global Burden of Disease, Injury, and Risk Factor Study 2016 (GBD 2016), we aimed to describe the burden of disease attributable to depressive disorders in terms of prevalence and disability-adjusted life years (DALYs) in South Asia countries (namely India, Pakistan, Bangladesh, Nepal and Bhutan).

**Methods:**

GBD 2016 used epidemiological data on depressive disorders (major depression and dysthymia) from South Asia and a Bayesian meta-regression tool (DisMod-MR 2.1) to model prevalence and DALYs of depressive disorders by age, sex, country and year. DALYs were calculated from the years lived with disability (YLDs), derived from the prevalence of depressive disorders and disability weights, obtained from a community and internet-based surveys. The analyses adjusted for comorbidity, data sources and multiple modelling, and estimates were presented with 95% uncertainty intervals (UI).

**Results:**

In 2016, the age-standardised prevalence of depressive disorders in South Asia was 3.9% (95% UI: 3.6–4.2%), 4.4% (95% UI: 4.4–4.8%) in Bangladesh, 3.9% (95% UI: 3.6–4.2%) in India, 3.0% (95% UI: 2.8–3.3%) in Pakistan, 4.0% (95% UI: 3.7–4.3%) in Nepal and 3.7% (95% UI: 3.4–4.1%) in Bhutan. In South Asia, depressive disorders accounted for 9.8 million DALYs (95% UI: 6.8–13.2 million) or 577.8 (95% UI: 399.9–778.9) per 100,000 population in 2016. Of these, major depressive disorders (MDD) accounted for 7.8 million DALYs (95% UI: 5.3–10.5 million). India generated the largest numbers of DALYs due to depressive disorders and MDD, followed by Bangladesh and Pakistan. DALYs due to depressive disorders were highest in females and older adults (75–79 years) across all countries.

**Conclusion:**

Our findings show the substantial public health burden of depressive disorders in South Asian populations and healthcare systems. Given the scale of depressive disorders, improvement in overall population health is possible if South Asian countries prioritise the prevention and treatment of depressive disorders.

**Electronic supplementary material:**

The online version of this article (10.1186/s12888-018-1918-1) contains supplementary material, which is available to authorized users.

## Background

Depressive disorders are one of the most common mental health illnesses worldwide, affecting approximately 121 million people and accounting for 5% of disability-adjusted life years (DALYs) globally [1, 2]. A previous global study suggested that major depressive disorder (MDD) would become the second leading cause of disease burden by 2020, after heart disease [3]. South Asia represents approximately 23% of the global population and one-fifth of the world’s mental health cases [4]. In the past five decades, significant advances have been made in the management of depressive disorders, including the development of various pharmacological and non-pharmacological interventions that have improved patients outcomes [5, 6]. However, the higher cost of drugs, prolonged use of medications and regular clinician appointments have been shown to be impediments to pharmacological and psychotherapeutic treatments of depressive disorders in many communities in South Asia and may have led people with depressive disorders to seek alternative therapies [7, 8].

Previous studies of MDD based on the diagnostic criteria reported that the proportion of people with MDD in South Asian countries was among the highest globally [4, 9]. A recent population-based mental health survey based on case definitions of mental disorders from India indicated that the prevalence of MDD was approximately 3.0% [10], substantially lower than estimates (21–83%) from other small-scale studies which assessed MDD with symptom scales [11–13]. Past studies which also used symptom scales in other South Asian countries also reported higher figures, 46% in urban Pakistan [14], 29% in regional Bhutan [15] and rural Bangladesh [16], respectively. In many developing countries (including South Asia), there is an increasing societal awareness and better acknowledgement of mental illness [10, 17, 18]. This increasing recognition of mental disorders in many communities has led to global, regional and national efforts to address the burden of mental illness, including depressive disorders [19, 20].

Depressive disorders are associated with adverse health outcomes and reduced life-expectancy, including chronic diseases such as diabetes, arthritis, coronary heart disease and cancers [6], and may lead to poor maternal and perinatal health outcomes, disruptions of family relationships [21, 22], poor work performance, physical inactivity, increased risk of self-injury, substance abuse and adverse life events (such as suicide) [23, 24]. To scale up and prioritise interventions to promote positive mental health requires an understanding of how mental illness differs across population groups and geographies [25]. Specifically, detailed knowledge of the burden of depressive disorders is essential to inform policy decision-making and health advocacy to ensure the development and/or revision of mental health programmes to improve population health outcomes.

The Global Burden of Disease, Injury and Risk Factor (GBD) study provides comprehensive and comparable health data for 195 countries and territories to facilitate timely and policy decision-making processes. In many instances, detailed expositions of the GBD findings for various health focus areas and geographies are further highlighted in additional publications to better characterise the results for researchers, clinicians and/or policy decision-makers to identify high-priority populations, as well as assess where resources could be best allocated [9, 26–29]. Therefore, an up-to-date assessment of the burden of depressive disorders focused in South Asian countries is essential for decision-makers and health professionals in the region to inform strategic mental health actions. The present study aimed to describe the prevalence and disability-adjusted life years (DALYs) associated with depressive disorders by country, sex and age in the South Asian countries of India, Pakistan, Bangladesh, Nepal and Bhutan, using data from the most recent GBD 2016 study. DALYs are a population health measure of overall disease burden, defined as the number of years lost due to ill-health, disability or premature death. DALYs have been used previously for priority-setting and resource allocation in health care delivery [30, 31].

## Method

The GBD study provides estimates of incidence, prevalence, cause of morbidity and mortality, and health loss, by age, sex, year, location, income group and overtime for diseases, injuries and key risk factors. The GBD study is a signatory to the Guidelines for Accurate and Transparent Health Estimates Reporting (GATHER) protocol. GATHER promotes good practice and transparency in reporting health estimates and permits researchers and decision-makers to assess the quality of the estimates [32]. The overall conceptual and analytical framework for the calculation of prevalence and DALYs for depressive disorders have been described in detail elsewhere [2, 33.]. In this study, we provide an overview of the methodology used to acquire relevant epidemiologic data and the estimation process of depressive disorders in South Asia.

### Case definition

In GBD 2016, depressive disorders included MDD and dysthymia. Depressive disorders were defined based on the criteria proposed in the Diagnostic and Statistical Manual of Mental Disorders-IV (DSM-IV) and the International Classification of Diseases 10 (ICD-10) [33]. The DSM-IV and ICD-10 definitions of the depressive disorders as described in this study are published in GBD 2015 and 2016 nonfatal health outcome papers [33, 34] and supplement [35]. The DSM-IV (296.21–24, 296.31–34) describes MDD as an episodic disorder with a prolonged outcome and an increased risk of death [36], comparable to ICD-10’s description of recurrent depressive disorder (F32.0–9, F33.0–9) [37]. MDD is the presence of at least one major depressive episode (i.e., an experience of depressed mood almost all day, every day, for at least 2 weeks). Dysthymia is a less severe depressed mood compared to MDD, lasting several years, with low rates of remission and no increased risk of death (DSM-IV: 300.4 [36]; ICD-10: F34.1 [37]).

### Data sources

The GBD 2016 study used prevalence, incidence, remission or duration, and excess mortality data from a systematic review employed for GBD 2010 [38], and additional reviews conducted between January 2013 and September 2016 to generate current estimates [2]. The reviews involved electronic searches of the peer-reviewed literature (PsycINFO, Embase and PubMed), the grey literature, expert consultations, as well as institutional collaborations, including the partnership between the GBD study team and the Indian Council of Medical Research and the Public Health Foundation of India [33]. The search yielded twenty-seven sources of epidemiological data for South Asian countries which were used for the estimation of prevalence and DALYs in those countries (Additional file 1: Table S1) [2, 33].

The inclusion criteria for the reviews included: (i) studies which reported prevalence, incidence, duration and/or excess mortality data from 1980 onward; (ii) “caseness” based on clinical threshold, consistent with the DSM or ICD criteria; (iii) detailed information must be provided on the study method and sample characteristics to assess the study quality; and (iv) study samples must be representative of the general population. Studies which reported information on an inpatient or pharmacological treatment, case studies, veterans or refugee samples were excluded [2].

Data sources used for the estimation of depressive disorders in GBD 2016 for South Asian countries can be accessed in the Global Health Data Exchange website (http://ghdx.healthdata.org/gbd-2016/data-input-sources). GBD 2016 provides up-to-date prevalence and DALYs data of depressive disorders based on country-specific epidemiologic data in the global health data exchange repository (GHDx) and improvements in methodological approaches to GBD 2010 [39], 2013 [40] and 2015 [34] studies. The GHDx provides researchers, clinicians and policy-makers access to GBD 2016 input sources, results and creates opportunities for discussing population health based on the best available data. GHDx also raises awareness about different groups collecting data globally and provides standardised citations to encourage appropriate acknowledgement of data owners’ contributions [41].

### Data analysis

In the estimation of each depressive disorder, epidemiological data were generated using DisMod-MR 2.1 – a Bayesian meta-regression tool. DisMod-MR 2.1 was the primary method used in GBD 2016 to combined all data sources, and adjust for variability and study quality to estimate the epidemiology of depressive disorders. Country-specific epidemiological data on depressive disorders from the systematic reviews were modelled in DisMod-MR 2.1 to estimate prevalence and DALYs by age and year, which also propagated uncertainty around the raw data through to the final estimates [2, 33]. Additionally, study- and location-level covariates were used to accommodate between-study variability and to indicate a positive association between conflict status and the proportion of depressive disorders, respectively [2].

In the GBD, DALYs are calculated by summing years lived with disability (YLDs) and years of life lost (YLLs) for each country, sex, age and year, wherein they measure the gap between the health of a population and the maximum lifespan spent in full health [33]. YLLs are calculated by multiplying the number of deaths attributable to the particular disorder at a specific age by the standard life expectancy for that age. [33]. In the present analysis, however, the YLL component was not calculated given that depression is not a cause of death in GBD study, consistent with the ICD-10 criteria for categorical designation of the cause of death to a single underlying cause [2]. Thus, DALYs were estimated for depressive disorders from the YLD component. YLDs were defined as the years of life lived with any short-term or long-term health loss, and were calculated by multiplying depression-specific prevalence data by disability weights [33]. Disability weights are population assessment of severity of health loss from a specific cause. GBD 2016 used disability weights, obtained from multi-country population-based surveys, where individuals were asked to indicate which person they perceived to be in good health, ranging from ‘perfect health’, coded as ‘0’ to death, coded as ‘1’ [2].

YLDs were adjusted for comorbidity given that individuals can have more than one disease at a particular time. Microsimulations were performed to assess individual comorbidity, and a multiplicative method was employed to estimate the combined disability experience among individuals with multiple diseases [2]. The rationale for comorbidity adjustment is that it estimates the difference between the mean disability weight in people with one disease and combined disability weight in those with more than one disease. This analytical strategy was similar to the approaches used in GBD 2013 [9] and 2015 [34].

To determine the proportion of people within each levels of severity for a depressive disorder, data from the US National Epidemiological Survey on Alcohol and Related Conditions (NESARC, conducted between 2001 and 2005) and the Australian National Survey of Mental Health and Wellbeing of Adults (NSMHWB, conducted in 1997) were used to estimate the proportions of MDD and dysthymic cases, categorised as asymptomatic and symptomatic (mild, moderate and severe) [2]. This approach captures the main features of MDD and dysthymia based on the DSM-IV and ICD-10 criteria, consistent with methodologies employed in the previous GBD studies [9, 34].

Corresponding 95% uncertainty intervals (UIs) for prevalence and DALYs estimates were generated from 1000 draws from the posterior distribution of each estimation process [33]. Unlike confidence intervals, UIs do not only adjust for sampling error but also capture uncertainty from several analytical modelling stages and adjust for type and quality of data sources [33].

## Results

### Prevalence of depressive disorders

In 2016, the age-standardised prevalence of depressive disorders was 3.9% (95% UI: 3.6–4.2%) in South Asia. In Bangladesh, age-standardised prevalence was 4.4% (95% UI: 4.4–4.8%), 3.9% (95% UI: 3.6–4.2%) in India, 3.0% (95% UI: 2.8–3.3%) in Pakistan, 4.0% (95% UI: 3.7–4.3%) in Nepal and 3.7% (95% UI: 3.4–4.1%) in Bhutan in the same year (Table 1).

**Table 1:** Age-standardised prevalence and absolute rate of DALYs for depressive disorders in South Asia, 1990–2016

		% (95% UI)	1990 rate^¥^ (95% UI)	2016 rate^¥^ (95% UI)	% change of DALYs rate 1990–2016
Depressive disorders	South Asia	3.9 (3.6–4.2)	529.5 (366.4–718.7)	577.8 (399.9–778.9)	9.1
	India	3.9 (3.6–4.2)	536.3 (370.8–729.4)	581.1 (403.6–785.2)	8.3
	Pakistan	3.0 (2.8–3.3)	434.3 (298.1–592.6)	475.0 (327.7–643.6)	9.4
	Bangladesh	4.4 (4.4–4.8)	583.0 (405.6–787.4)	680.6 (467.3–925.6)	16.7
	Nepal	4.0 (3.7–4.3)	466.3 (321.4–633.8)	534.7 (366.9–725.7)	14.7
	Bhutan	3.7 (3.4–4.1)	453.6 (314.9–619.3)	530.3 (361.6–716.3)	16.9
Major depressive disorder	South Asia	2.6 (2.4–2.8)	430.7 (297.5–585.1)	457.4 (314.5–617.5)	6.2
	India	2.5 (2.3–2.8)	436.2 (300.6–592.3)	458.5 (315.8–620.9)	5.1
	Pakistan	2.3 (2.1–2.6)	341.7 (233.5–470.9)	367.0 (251.3–502.7)	7.4
	Bangladesh	3.1 (2.8–3.5)	488.9 (335.3–664.5)	561.7 (382.4–763.5)	14.9
	Nepal	2.6 (2.1–2.9)	371.5 (254.8–508.7)	424.4 (288.0–573.4)	14.3
	Bhutan	2.4 (2.2–2.7)	361.3 (248.7–495.4)	412.6 (281.0–557.6)	14.2
Dysthymia	South Asia	1.4 (1.2–1.6)	98.8 (66.6–143.8)	120.4 (81.2–174.1)	21.9
	India	1.4 (1.2–1.6)	100.2 (67.6–145.2)	122.6 (82.5–177.2)	22.4
	Pakistan	1.4 (1.2–1.6)	92.7 (62.6–135.6)	108.0 (72.7–156.5)	16.5
	Bangladesh	1.4 (1.2–1.6)	94.2 (63.2–138.0)	118.9 (79.9–174.4)	26.3
	Nepal	1.4 (1.2–1.6)	94.9 (64.1–139.6)	110.3 (74.4–159.6)	16.3
	Bhutan	1.4 (1.2–1.6)	92.2 (62.4–133.7)	117.8 (78.9–171.8)	27.7

%: prevalence; ^¥^rate is per 100,000 population; *UI* uncertainty interval, *DALYs* disability-adjusted life years

### YLDs and DALYs due to depressive disorders

In 2016, depressive disorders contributed 577.8 (95% UI: 399.9–778.9) per 100,000 population in South Asian countries, an increase of 9% (Table 1) or a total of 9.8 million DALYs (95% UI: 6.8–13.2 million) in 2016 (Table 2). The majority of DALYs (7.8 million, 95% UI: 5.3–10.5 million) were due to MDD. In South Asia, MDD accounted for 2% (95% UI: 1–3%) of the total cause of DALYs in 2016 for both sexes and all age groups and was ranked the 19th leading cause of disease burden – an increase from a ranking of 30th leading cause of disease burden in 1990, data not shown.

**Table 2:** Numbers of disability-adjusted life years by depressive disorders in South Asia, 1990–2016

		1990	2016	% change of DALYs numbers 1990–2016
		DALYs (95% UI)	DALYs (95% UI)	
Depressive disorders	South Asia	5,800,499 (4,014,423-7,873,652)	9,821,063 (6,797,636-1323,9210)	69.3
	India	4,632,923 (3,202,901-6,300,340)	7,647,086 (5,311,795-10,332,886)	65.1
	Pakistan	470,327 (322,832-641,750)	907,016 (625,699-1,228,954)	92.8
	Bangladesh	607,410 (422,605-820,278)	1,101,909 (756,521-1,498,522)	81.4
	Nepal	87,429 (60,251-118,821)	160,820 (110,353-218,278)	83.9
	Bhutan	2410 (1673-3291)	4232 (2885-5716)	75.6
Major depressive disorder	South Asia	4,718,648 (3,259,338-6,410,444)	7,774,678 (5,345,086-1,0496,504)	64.8
	India	3,767,801 (2,596,567-5,116,562)	6033,551 (4,155,478-8,171,522)	60.1
	Pakistan	369,982 (252885–509,986)	700,813 (479,865-959,981)	89.4
	Bangladesh	509,304 (349,326-692,269)	909,375 (619,061-1,236,065)	78.6
	Nepal	69,641 (47,763-95,369)	127,647 (86,607-172,470)	83.3
	Bhutan	1920 (1321-2632)	3292 (2242-4449)	71.5
Dysthymia	South Asia	1,081,851 (729,209-1,575,227)	2,046,386 (1,380,766-2,958,677)	89.2
	India	865,123 (584,292-1,254,327)	1,613,535 (1,086,278-2,332,034)	86.5
	Pakistan	100,345 (67,839-146,800)	206,203 (138,778-298,937)	105.5
	Bangladesh	98,106 (65,798-143,768)	192,534 (129,339-282,364)	96.3
	Nepal	17,787 (12,016-26,176)	33,173 (22,367-48,012)	86.5
	Bhutan	490 (331–711)	940 (629–1371)	91.7

*DALYs* disability-adjusted life years, *UI* uncertainty interval

India had the most substantial numbers of DALYs for all depressive disorders in 2016 at 7.7 million DALYs (95% UI: 5.3–10.3 million), accounting for more than two-thirds of the DALYs in South Asia. Bangladesh followed with 1.1 million DALYs (95% UI: 756,521–1.5 million) and Pakistan at 907,016 DALYs (95% UI: 625,699–1.2 million). Similarly, India had the highest numbers of DALYs for MDD at 6.1 million DALYs (95% UI: 4.2–8.2 million), followed by Bangladesh at approximately 1 million DALYs (95% UI: 619,061–1.2 million) (Table 2).

**Fig. 1:**
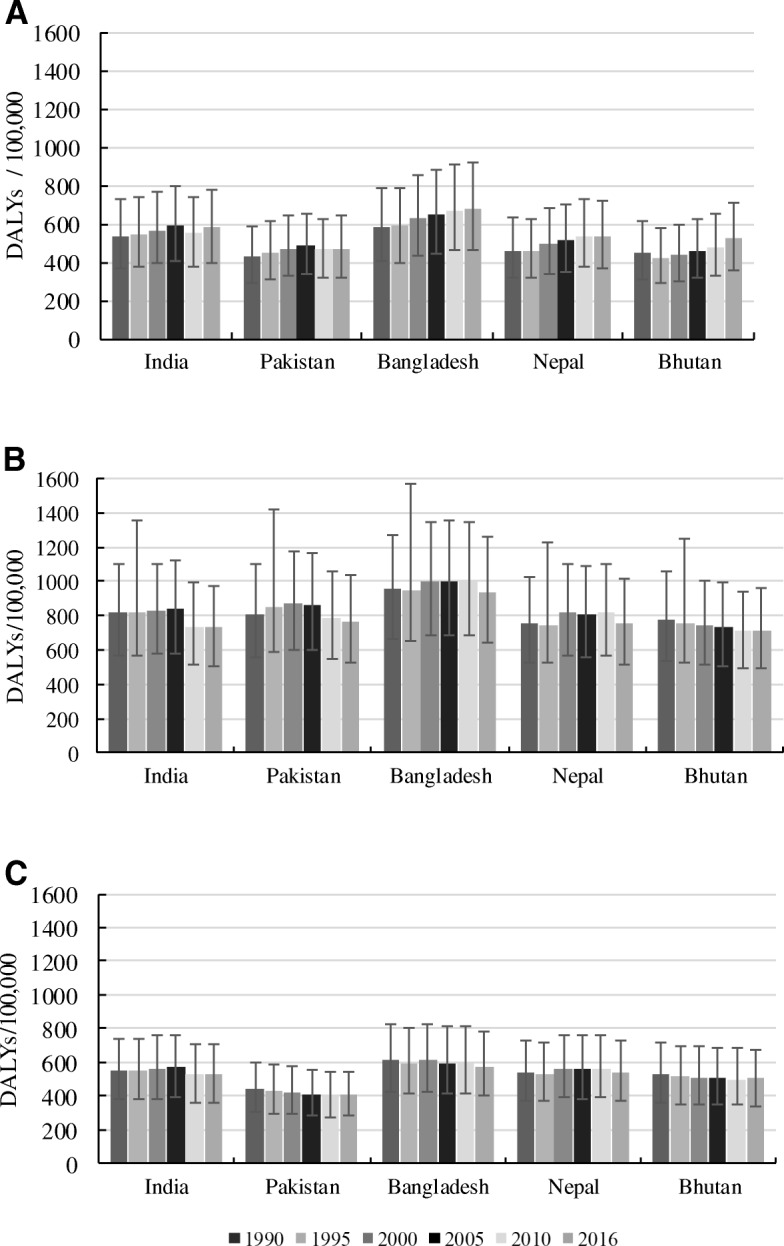
Age-standardised rate of DALYs due to depressive disorders by sex in South Asia, 1990–2016. **a** Both sexes; (**b**) Females; (**c**) Males

**Fig. 2:**
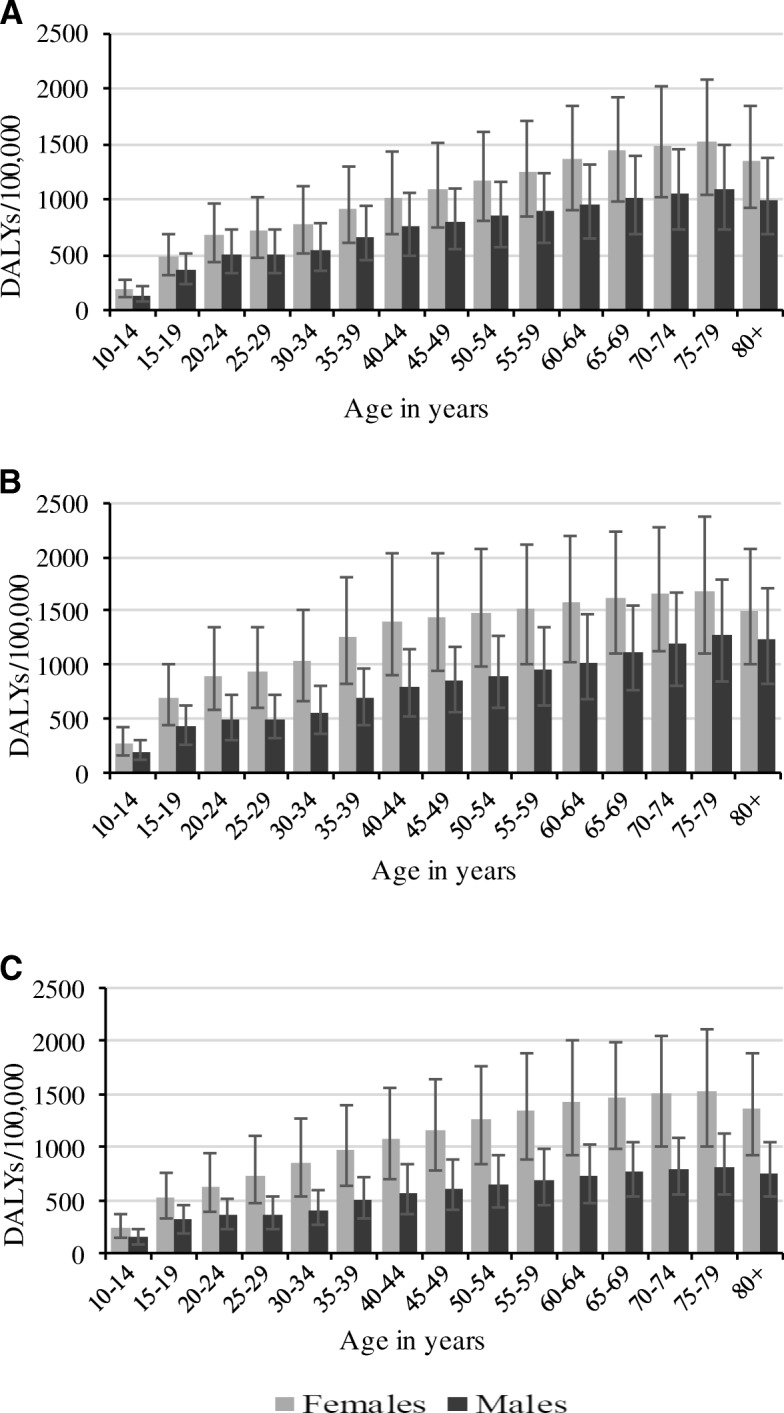
Rate of DALYs due to depressive disorders by age in India, Bangladesh and Pakistan, 2016. **a** India; (**b**) Bangladesh; (**c**) Pakistan

**Fig. 3:**
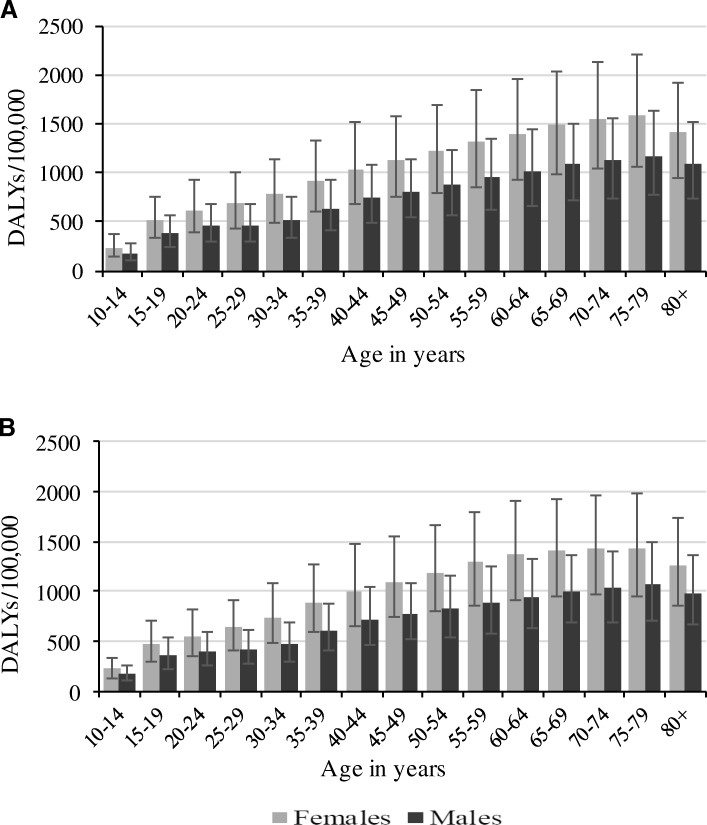
Rate of DALYs due to depressive disorders by age in Nepal and Bhutan in 2016. **a** Nepal; (**b**) Bhutan

Figure 1 shows the age-standardised rate of depressive disorders by sex and country. Between 1990 and 2016, higher rates of DALYs were evident in all South Asian countries. Over the same period, DALYs rates were relatively higher in females compared to their male counterparts in South Asia, but this was not statistically significant. A stratified analysis of the rate of DALYs by age groups showed that the burden of depressive disorders was highest among older age groups (75–79 years) in all countries (Figs. 2 and 3). Between 1990 and 2016, age-standardised YLDs were highest in Bangladesh for both sexes, with higher levels seen among females compared to males but this was not statistically significant (Additional file 2: Figure S1).

## Discussion

The global prevalence of depressive disorders in 2016 was 3.7% [2], which is comparable to the reported age-standardised prevalence of 3.9% in South Asia. MDD was the largest contributor to the total burden of depressive disorders in all South Asian countries. Similarly, MDD ranks first among other mental and substance use disorders, and sixth in all diseases and injuries in the GBD 2016 study [1]. Age-standardised rate of DALYs was largest in Bangladesh, with a higher burden in females and older adults but this was not statistically significant.

The current study showed that MDD accounted for the largest proportion and numbers of DALYs due to depressive disorders in South Asia. This finding is consistent with a previous study which found that the majority of depressive disorders worldwide related to MDD [9]. The previous study also suggested that the burden of depressive disorders was higher among high-income North American and European countries compared to Asian countries [9]. Possible reasons for this finding may be due to the under-reporting of mental illness or a lack of high-quality epidemiological data on mental health disorders in many developing countries [9, 42]. Under-reporting of depressive disorders in the South Asian region may also be partly due to a high level of stigma and discrimination associated with mental health disorders in South Asian countries [43–47].

A study conducted in Nepal indicated that stigma and discrimination were the most common factors associated with a lack of uptake of mental health treatment, where self-disclosure of traumatic events is socially stigmatised. Additionally, people believe that disclosing one’s traumatic experience may affect the reputation of a person and their family [48]. Since 2006, the Government of Nepal introduced the Mental Health Act to improve the mental health outcomes of the population [49], including the involvement of medical colleges to strengthen collaboration between researchers and community stakeholders [50]. However, mental health initiatives in Nepal remain under-resourced as only 0.1% of the national health budget is allocated to mental health interventions [49]. To promote the mental health of the Nepalese population, government at all levels in Nepal would have to increase funding for mental health programmes and be committed to supporting evidence-based and culturally-appropriate interventions.

The study showed that Bangladesh had the highest age-standardised prevalence of MDD, and it was one of the leading contributors to the burden of depressive disorders in the South Asian region, consistent with previous studies [9, 17] and a World Health Organisation (WHO) report [51]. In Bangladesh, there are efforts to reduce the burden of mental health disorders. For example, between 2005 and 2006, the Government of Bangladesh in collaboration with the WHO and primary health sector introduced programmes to create awareness and educate people on mental illness, increased the number of mental health professionals, as well as developed research capacities [51]. However, improvement in mental health funding will be required given that Bangladesh – where over 65% of health expenditures is out-of-pocket – recently allocated only 0.5% of the total health budget to mental health initiatives [52].

In South Asian countries, depressive disorders are more common among people from lower socio-economic background, those with no or lower education, those unemployed, divorced or widowed (especially women), and the elderly [10, 53–56]. Consistent with previous reports [9, 57, 58], the present study showed that the burden of depressive disorders was higher among females and older adults compared to males and young people, respectively, in all South Asian countries. Past studies have reported that compared to males, females are more likely to experience adverse life events that are strongly associated with the onset of depressive episodes, including gender discrimination, physical and sexual abuse, relationship breakdown, intimate partner violence, antenatal and postnatal stress, and adverse cultural norms [59–61]. There is limited evidence to attribute a hormonal or biological mechanism as a potential explanation for the higher burden of depressive episodes among women than men [62]. To improve mental health outcomes in South Asia, mental health interventions should target the general population, particularly females and those in vulnerable environments.

The present study showed that the age-standardised prevalence of MDD and dysthymia was 2.5% and 1.4%, respectively in India, in line with previous reports [9, 10]. However, studies from regional areas of India have indicated that the prevalence of MDD ranged from 21 to 83% [11–13]. This variation is due to differences in case definitions and the measurement of depressive episodes, wherein those small-scale studies assessed MDD with symptom scales. Since the 1970s [63, 64], India has taken necessary steps to address mental health issues, including increasing funding for mental health programmes, improving institutional care and research, and motivating relevant stakeholders to implement mental health laws at all levels of government [65, 66]. However, the shortages of healthcare professionals and stigma associated with mental health illness remain an obstacle to the uptake of relevant health care measures in the country [67–69]. Task-sharing (such as training of non-physicians) to provide focused and relevant health services in addition to health care workers in communities, designing and promoting life skills and family education programmes have been advocated to improve mental health outcomes in India [65, 67].

Among the South Asian countries studied, Pakistan had a lower prevalence of depressive disorders at 3.0%, which may be attributable to under-reporting and a lack of high-quality epidemiologic data. Previous reports suggested that weakening of traditional values and family systems due to urbanisation, fear of wars and natural disasters may be associated with an increasing burden of depressive disorders in Pakistan [47, 70]. The prevalence of depressive disorders in Bhutan was 3.7%, with an associated higher rate of DALYs attributable to depressive disorders. Like the Pakistanis [47], Bhutanese people often believe that ‘Karma’ (previous life) and attitudes strongly influence a person’s propensity to experience mental illness. Consequently, more than 99% of Bhutanese with mental illness seek alternative therapies such as faith and religious healing [71].

In the last decade, Bhutan has introduced a number of mental health initiatives to increase accessibility and availability of primary mental health services, with a subsequent increase in mental health awareness [71, 72]. These efforts have been revised to incorporate other key measures (such as ‘National Happiness Index’) to promote the well-being of individuals and inform policy decision-making [25]. Nevertheless, improvements are still needed in Bhutan as the country has no trained psychiatrists in community-based mental health programmes [71]. For Pakistan, the mental health strategy has multiple weaknesses, including inadequate funding and a lack of service integration [14, 73]. Training of health workers, development of research capacity and epidemiological data collection, and refinement of current mental health programmes, as well as an increase in mental health financing, galvanised with strong political will at all levels, are warranted in those countries [15, 72].

The study has a number of limitations, which have been described in detail in the Lancet Series for the GBD and supplement [2, 9, 33]. Briefly, we describe specific limitations. First, DSM-IV and ICD-10 criteria for depressive disorders may not be applicable across all cultural sub-population groups in South Asia as people prefer not to disclose mental health issues as a result of stigma and discrimination. Second, GBD study used disability weights to capture health loss while not accounting for the impact of depressive disorders on economic productivity and family socio-emotional interaction. Third, the lack of assessment of YLLs for depressive disorders presents challenges in the full estimation of health loss due to depressive disorders as they are not coded as causes of death in the GBD study based on the ICD-10 criteria for listing causes of death. Fourth, it is possible that the study may have underestimated the ‘full burden’ of depressive disorders given data availability and a time lag between the in-country release of data and their subsequent incorporation into the GBD study. The lack of high-quality epidemiological data for all countries is possibly reflected in the wide uncertainty intervals. However, underestimation of data may be less likely in India given gbd 2016 access to recent epidemiological data and improvements in methodological and modelling strategies.

## Conclusion

The study indicates that the prevalence of depressive disorders in South Asia is comparable to the global estimate, and Bangladesh and India has higher proportions of people with depressive disorders in South Asia. Additionally, females and older adults (75–79 years) have the highest burden of depressive disorders across all countries in the region. The findings suggest that mental health services and programmes should be prioritised and scaled up across South Asian countries, with significant contributions and involvement of national and subnational governments to improve population health and well-being.

## Additional file

Additional file 1: **Table S1.** Data sources used for estimating the burden of depressive disorders in South Asian countries. (DOCX 18 kb)

Additional file 2: **Figure S1.** Age-standardised rate of YLDs due to depressive disorders by sex in South Asia, 1990–2016. (A) Both sexes; (B) Females; (C) Males. (DOCX 30 kb)

## Abbreviations

DALYs: Disability-adjusted life years
GBD: Global Burden of Disease
YLDs: Years lived with disability
YLLs: Years of life lost
