# Supplementary material for: The burden of depressive disorders in South Asia, 1990–2016: findings from the global burden of disease study
Source: BMC Psychiatry. 2018 Oct 16;18:333. doi: 10.1186/s12888-018-1918-1 (PMC6192293; doi:10.1186/s12888-018-1918-1)
Supplement: Supplementary file 1 — Table S1. Data sources used for estimating the burden of depressive disorders in South Asian countries. (DOCX 18 kb) [file 12888_2018_1918_MOESM1_ESM.docx]

**Table S1 Data sources used for estimating the burden of depressive disorders in South Asian countries**

| **Serial No** | **Citation** |
| --- | --- |
|  | World Health Organization (WHO). Bangladesh World Health Survey 2003. Geneva, Switzerland: World Health Organization (WHO), 2005 |
|  | International Institute for Population Sciences (India), World Health Organization (WHO). India World Health Survey 2003. Geneva, Switzerland: World Health Organization (WHO), 2005 |
|  | Srinath S, Girimaji SC, Gururaj G, Seshadri S, Subbakrishna DK, Bhola P, Kumar N. Epidemiological study of child and adolescent psychiatric disorders in urban and rural areas of Bangalore, India. Indian J Med Res. 2005; 122(1): 67-79 |
|  | Ahmad A, Khalique N, Khan Z, Amir A. Prevalence of psychosocial problems among school going male adolescents. Indian J Community Med. 2007; 32(3): 219-21 |
|  | Bansal PD, Barman R. Psychopathology of school going children in the age group of 10-15 years. Int J Appl Basic Med Res. 2011; 1(1): 43-7 |
|  | Patil RN, Nagaonkar SN, Shah NB, Bhat TS. A Cross-sectional Study of Common Psychiatric Morbidity in Children Aged 5 to 14 Years in an Urban Slum. J Fam Med Prim Care. 2013; 2(2): 164-8 |
|  | Poongothai S, Pradeepa R, Ganesan A, Mohan V. Prevalence of depression in a large urban South Indian population – the Chennai Urban Rural Epidemiology Study (CURES-70). PLoS One. 2009; 4(9): e7185 |
|  | Rajkumar AP, Thangadurai P, Senthilkumar P, Gayathri K, Prince M, Jacob KS. Nature, prevalence and factors associated with depression among the elderly in a rural south Indian community. Int Psychogeriatr. 2009; 21(2): 372-8 |
|  | Sarkar S, Sinha VK, Praharaj SK. Depressive disorders in school children of suburban India: an epidemiological study. Soc Psychiatry Psychiatr Epidemiol. 2012; 47(5): 783-8 |
|  | Silvanus V, Subramanian P. Epidemiological study of mental morbidity in an urban slum community in India for the development of a community mental health programme. Nepal Med Coll J. 2012; 14(1): 13-7 |
|  | Kessler RC, Birnbaum HG, Shahly V, Bromet E, Hwang I, McLaughlin KA, Sampson N, Andrade LH, de Girolamo G, Demyttenaere K, Haro JM, Karam AN, Kostyuchenko S, Kovess V, Lara C, Levinson D, Matschinger H, Nakane Y, Browne MO, Ormel J, Posada-Villa J, Sagar R, Stein DJ. Age differences in the prevalence and co-morbidity of DSM-IV major depressive episodes: results from the WHO World Mental Health Survey Initiative. Depress Anxiety. 2010; 27(4): 351–64 |
|  | Sathyanarayana Rao TS, Darshan MS, Tandon A, Raman R, Karthik KN, Saraswathi N, Das K, Harsha GT, Krishna VST, Ashok NC. Suttur study: An epidemiological study of psychiatric disorders in south Indian rural population. Indian J Psychiatry. 2014; 56(3): 238–45 |
|  | Guerra M, Prina AM, Ferri CP, Acosta D, Gallardo S, Huang Y, Jacob KS, Jimenez-Velazquez IZ, Llibre Rodriguez JJ, Liu Z, Salas A, Sosa AL, Williams JD, Uwakwe R, Prince M. A comparative cross-cultural study of the prevalence of late life depression in low and middle income countries. J Affect Disord. 2016; 190: 362–8 |
|  | Shidhaye R, Gangale S, Patel V. Prevalence and treatment coverage for depression: a population-based survey in Vidarbha, India. Soc Psychiatry Psychiatr Epidemiol. 2016; 51(7): 993–1003 |
|  | Mathias K, Goicolea I, Kermode M, Singh L, Shidhaye R, Sebastian MS. Cross-sectional study of depression and help-seeking in Uttarakhand, North India. BMJ Open. 2015; 5(11): e008992 |
|  | Behera P, Sharan P, Mishra AK, Nongkynrih B, Kant S, Gupta SK. Prevalence and determinants of depression among elderly persons in a rural community from northern India. Natl Med J India. 2016; 29(3): 129–35 |
|  | Ministry of Health and Family Welfare (India), National Institute of Mental Health and Neurosciences (India), World Health Organization (WHO). India National Mental Health Survey 2015-2016 |
|  | Ministry of Health and Family Welfare (India), National Institute of Mental Health and Neurosciences (India), World Health Organization (WHO). India National Mental Health Survey Data Tables 2015-2016 |
|  | World Health Organization (WHO). Nepal World Health Survey 2003. Geneva, Switzerland: World Health Organization (WHO), 2005 |
|  | Subedi S, Tausig M, Subedi J, Broughton CL, Williams-Blangero S. Mental illness and disability among elders in developing countries: the case of Nepal. J Aging Health. 2004; 16(1): 71-87 |
|  | Kohrt BA, Hruschka DJ, Worthman CM, Kunz RD, Baldwin JL, Upadhaya N, Acharya NR, Koirala S, Thapa SB, Tol WA, Jordans MJD, Robkin N, Sharma VD, Nepal MK. Political violence and mental health in Nepal: prospective study. Br J Psychiatry. 2012; 201(4): 268-75 |
|  | Luitel NP, Jordans MJD, Sapkota RP, Tol WA, Kohrt BA, Thapa SB, Komproe IH, Sharma B. Conflict and mental health: a cross-sectional epidemiological study in Nepal. Soc Psychiatry Psychiatr Epidemiol. 2013; 48(2): 183-93 |
|  | World Health Organization (WHO). Pakistan World Health Survey 2003-2004. Geneva, Switzerland: World Health Organization (WHO), 2005 |
|  | Nisar N, Billoo N, Gadit AA. Prevalence of depression and the associated risks factors among adult women in a fishing community. J Pak Med Assoc. 2004; 54(10): 519-25 |
|  | Mumford DB, Minhas FA, Akhtar I, Akhter S, Mubbashar MH. Stress and psychiatric disorder in urban Rawalpindi. Community survey. Br J Psychiatry. 2000; 557-62 |
|  | Mumford DB, Nazir M, Jilani FU, Baig IY. Stress and psychiatric disorder in the Hindu Kush: a community survey of mountain villages in Chitral, Pakistan. Br J Psychiatry. 1996; 168(3): 299-307 |
|  | Mumford DB, Saeed K, Ahmad I, Latif S, Mubbashar MH. Stress and psychiatric disorder in rural Punjab. A community survey. Br J Psychiatry. 1997; 473-8 |

(Source: <http://ghdx.healthdata.org/gbd-2016/data-input-sources>)
